# Effect of finerenone on urinary protein and inflammatory factor levels in type 2 diabetes mellitus patients with diabetic kidney disease

**DOI:** 10.1371/journal.pone.0329965

**Published:** 2025-08-18

**Authors:** Jia Song, Yan Kang, LiNa Wang, YuQing Guo, XinJu Jia, ShanShan Dong, YiMeng Li, Dan Wang, JingMin Gao, AiGe Yang

**Affiliations:** Department of Endocrinology, The First Hospital of Hebei Medical University, Shijiazhuang, Hebei, China; International University of Health and Welfare, School of Medicine, JAPAN

## Abstract

**Aims:**

This study aimed to investigate the efficacy and safety of finerenone in the treatment of diabetic kidney disease (DKD) in the real-world medical setting and explore the underlying mechanism of its kidney-protecting effects from the perspective of the inflammatory response.

**Materials and Methods:**

Forty-eight DKD patients were selected and completed a 6-month finerenone treatment. Renal parameters, inflammatory cytokines, other related indicators and adverse effects were collected at every visit. Additionally, subgroup analysis was conducted on the microalbuminuria group (baseline urinary albumin/creatinine ratio (UACR) 30 – < 300 mg/g) and the macroalbuminuria group (baseline UACR ≥ 300 mg/g).

**Results:**

After finerenone treatment, the levels of UACR, urinary β2-microglobulin (β2-MG) and proinflammatory cytokines in patients decreased compared with the pretreatment levels. Moreover, the rates of decrease in the UACR levels in the macroalbuminuria group were significantly greater than those in the microalbuminuria group. In the initial stage of treatment, the patient’s estimated glomerular filtration rate (eGFR) level decreased and serum potassium level increased compared to before, but with prolongation of the treatment time, both eGFR and serum potassium levels remained stable.

**Conclusions:**

With the assistance of RAS inhibitors, SGLT2 inhibitors, and GLP-1 receptor agonists, finerenone effectively reduces urinary protein and improves the inflammatory response, demonstrating relatively manageable safety in real-world DKD treatment. The patients with macroalbuminuria may experience greater benefits.

## 1. Introduction

Diabetic kidney disease (DKD) is a chronic kidney disease (CKD) that is caused by diabetes, which is the main cause of CKD in China. In recent decades, the morbidity and mortality of patients with DKD in China have significantly increased [1]. Even with standard treatment regimens, including renin‒angiotensin system inhibitors (RASi), the risk of DKD occurrence and progression remains high [2].

The overactivation of mineralocorticoid receptors (MR) plays an important role in the occurrence and development of DKD. The MR is widely expressed in renal cells, such as glomerular cells (including mesangial cells and podocytes), renal distal tubule cells and cortical collecting duct cells [3]. Taira M and other scholars have reported that the mRNA and protein expression of MR in the kidneys of diabetic rats is increased [4]. Additionally, the excessive binding of aldosterone to the MR in the kidneys can damage the kidneys through various mechanisms, such as by promoting proinflammatory and profibrotic factor expression [5,6]. The infiltration of many macrophages and increased expression of proinflammatory factors, such as interleukin-1β (IL-1β), tumour necrosis factor-α (TNF-α) can be observed in renal biopsy samples from DKD patients [7]. Previous studies have shown that the levels of serum proinflammatory factors such as tumour necrosis factor-α (TNF-α) and interferon-γ (IFN-γ) in diabetic nephropathy patients were higher than those in healthy subjects [8]. Moreover, various proinflammatory factors, such as IL-1 and TNF-α, damage the kidneys by increasing the permeability of renal endothelial cells and exerting cytotoxic effects [5]. The body can respond to this inflammatory response by activating anti-inflammatory pathways. For instance, interleukin-10 (IL-10) is a specific anti-inflammatory factor that can promote the expression of anti-inflammatory genes by binding to receptors on target cells, thereby inhibiting mesangial cell proliferation and delaying DKD progression [9].

The main clinical manifestations of DKD are increased urinary protein excretion and decreased estimated glomerular filtration rate (eGFR). Albuminuria is an ideal biomarker that is used in the clinic to evaluate kidney injury or kidney function in patients with DKD [10]. Additionally, β2-microglobulin (β2-MG), which is a molecule with a molecular weight of approximately 11,800 Da that is present on the surface of nucleated cells, undergoes glomerular filtration and is reabsorbed in the proximal tubules. However, when proximal renal tubular dysfunction occurs, the excretion of β2-MG in the urine increases accordingly. Thus, urinary β2-MG levels are a biomarker of renal tubular injury [11].

During the past decades, the understanding of the pathophysiology and treatment of DKD has progressed. Pharmaceutical drugs like RAS inhibitors, sodium-glucose cotransporter 2 inhibitors (SGLT2i) and glucagon-like peptide-1 receptor agonists (GLP-1RA) are mainly used in treating DKD. Despite the use of these drugs, there is still a residual risk of kidney disease progression and cardiorenal adverse outcomes in DKD [12,13]. Finerenone, a novel nonsteroidal MR antagonist (MRA), has been approved in many countries including the US, Europe, Japan, and China for the treatment of DKD. Finerenone is safer and more effective than first- and second-generation MRAs because of its higher selectivity and specificity. Finerenone binds to the MR through many van der Waals forces and hydrogen bonds, which explain its greater affinity and stronger antagonistic effects [14]. After binding to the MR, finerenone alleviates oxidative stress and reduces the downstream expression of proinflammatory and profibrotic factors, thereby delaying the progression of kidney diseases such as podocyte damage and disappearance, glomerular damage and tubulointerstitial fibrosis. Moreover, finerenone also protects against adipose tissue dysfunction, glucose metabolism disorders and cardiovascular dysfunction, thus ameliorating metabolic disorders that are associated with DKD [6,15].

Two randomized, double-blind, placebo-controlled, multicentre phase III trials, namely, FIDELIO-DKD and FIGARO-DKD, as well as the FIDELITY large-scale pooled analysis of these two trials, revealed that in type 2 diabetes patients with CKD, compared with placebo, finerenone reduces the risk of CKD progression and cardiovascular events and improves cardiovascular and renal prognosis [16–18]. At present, there are only a few studies on the efficacy and safety of finerenone in real-world clinical practice. This study aimed to investigate the safety and renal effect of finerenone in patients with DKD in routine clinical practice and explore the mechanism of finerenone in reducing urinary protein and protecting the kidney from the perspective of inflammatory response.

## 2. Materials and methods

### 2.1 Experimental design

This is a single center retrospective cohort study. This study collected the medical records of patients with diabetic nephropathy who visited the Department of Endocrinology of the First Hospital of Hebei Medical University from June 2023 to August 2024. The date of accessing the data is March 1st, 2025. The author can obtain information that can identify individual participants during data collection. All data were not fully anonymized before we accessed them.

### 2.2 Research subjects

Adult CKD patients (≥ 18 years old) with UACR ≥ 30 mg/g and eGFR ≥ 25 ml/min/1.73㎡ were screened. Inclusion criteria were: [1] patients diagnosed with DKD based on medical history and clinical presumption, or a pathological diagnosis suggesting diabetic nephropathy; [2] patients treated with finerenone according to the drug instruction; [3] patients with a follow-up period of no less than 6 months. Exclusion criteria were: [1] patients with concurrent active immune kidney disease, autosomal dominant polycystic kidney disease, or other causes of kidney disease; [2] patients treated with finerenone for no more than 3 months; [3] patients with incomplete follow-up data or who were lost to follow-up. Approval was obtained from the Clinical Research Ethics Committee of the First Hospital of Hebei Medical University. Approval number is [2024] Research and Approval No. (190). The participants provided their written informed consent to participate in this study.

### 2.3 Research methods

#### 2.3.1 Baseline data.

The age, sex, diabetes course, medication, blood pressure, UACR, urinary β2-MG, IL-1β, TNF-α, IFN-γ, IL-10, eGFR, serum potassium, HbA1c, triglyceride (TG), total cholesterol (TC), low-density lipoprotein cholesterol (LDL-C), alanine aminotransferase (ALT), aspartate aminotransferase (AST) and renal function-related indicator values of each subject were collected. The albumin and creatinine concentrations in the urine samples were measured with immunoturbidimetry and enzymatic methods, respectively (Beckman AU5800 fully automated biochemical analyser). The UACR was calculated by comparing the albumin concentration against the creatinine concentration; urinary β2-MG levels were analysed using latex-enhanced immunoturbidimetry (Beckman AU5800 fully automated biochemical analyser); IL-1β, TNF-α, IFN-γ, and IL-10 levels were analysed using flow cytometry (Navios flow cytometer); serum potassium, TG, TC, LDL-C, ALT, AST and renal function-related indicator levels were measured using enzymatic methods (Beckman LX20 fully automated biochemical analyser); and HbA1c levels were measured using the immune agglutination method (Siemens DCA2000 + HbA1c analyser and HbA1c kit). These tests were performed by dedicated personnel in strict accordance with the instructions. The eGFR was calculated using the CKD-EPI formula from the 2009 Chronic Kidney Disease Epidemiology Collaboration.

#### 2.3.2 Effectiveness indicators.

The UACR and the urinary β2-MG, IL-1β, TNF-α, IFN-γ, IL-10 levels were retrospectively collected after 3 and 6 months of treatment. Additionally, subgroup analysis was performed on the UACR levels and urinary β2-MG levels for the microalbuminuria group (baseline UACR 30 – < 300 mg/g) and the macroalbuminuria group (baseline UACR ≥ 300 mg/g).

#### 2.3.3 Safety indicators.

The eGFR and serum potassium level were retrospectively collected after 1 month, 3 months and 6 months of treatment with finerenone, and blood pressure and the ALT and AST levels were gathered after 3 and 6 months of treatment with finerenone. All the adverse events, including hyperkalaemia, hypotension, decreased eGFR, allergies, liver dysfunction, breast pain and male breast development, were recorded during the follow-up period.

#### 2.3.4 Other biochemical indicators.

HbA1c, TG, TC, and LDL-C levels were retrospectively collected after 3 and 6 months of treatment.

#### 2.3.5 Statistical analysis.

In this study, the Shapiro‒Wilk test was used to evaluate the normality of the quantitative data. The mean ± standard deviation (SD) was used to describe normally distributed data, and the median and interquartile range (IQR) were used to describe skewed data. In accordance with the results of the normality tests, paired t tests or Wilcoxon signed rank sum tests were used to compare the data before and after finerenone treatment, and Student’s t tests or Mann‒Whitney U tests were used to compare the data between subgroups.

## 3. Results

### 3.1 Baseline data

This study selected a total of 67 DKD patients treating with finerenone. Among them, 2 patients discontinued treatment with finerenone due to hyperkalemia resulting in less than 3 months of treatment, and 17 patients had incomplete or lost follow-up data. A total of 48 research subjects were included in the analysis for the study period. The initial oral dose of finerenone was 10 mg per day. After 6 months of treatment, 10 patients (20.8%) received a daily oral dose of 10 mg of finerenone, and 38 patients (79.2%) received a daily oral dose of 20 mg of finerenone. The patients’ demographic and clinical characteristics at baseline are shown in Table 1. At baseline, 54.2% of the patients received RAS inhibitors, 56.3% of the patients received SGLT2 inhibitors, and 33.3% of the patients received GLP-1 receptor agonists.

**Table 1:** Baseline characteristics of the participants.

Baseline characteristics	Total (n = 48)
Sex, male, n (%)	32 (66.7%)
Age (y)	53.81 ± 11.46
Duration of diabetes mellitus (y)	8.00 (5.00, 14.00)
Hypertension, n (%)	25 (52.1)
Abnormal blood lipid metabolism,n (%)	38 (79.2)
BMI (kg/m^2^)	26.03 ± 3.52
SBP (mmHg)	127.60 ± 9.06
DBP (mmHg)	81.77 ± 7.93
Baseline urinary protein indicators	
UACR (mg/g)	363.92 (126.90, 860.12)
UACR (30 – < 300 mg/g), n (%)	23 (47.9)
UACR (≥ 300 mg/g), n (%)	25 (52.1)
Urinary β2-MG (mg/L)	0.30 (0.11, 0.67)
Baseline biochemical indicators	
Creatinine (umol/l)	66.80 (55.25,92.43)
eGFR (ml/min/1.73㎡)	97.79(82.22, 111.13)
Serum potassium (mmol/l)	3.95 ± 0.34
Serum sodium (mmol/l)	138.15 (136.03,139.45)
HbA1c (%)	8.52 ± 1.96
TG (mmol/l)	2.17 (1.41, 2.96)
TC (mmol/l)	4.90 ± 1.27
LDL-C (mmol/l)	2.85 ± 0.93
ALT (u/L)	21.30 (16.18, 29.15)
AST (u/L)	19.75 (15.83, 29.00)
Combination medication	
Lipid lowering drugs, n (%)	35 (72.9)
RASi, n (%)	26 (54.2)
SGLT-2i, n (%)	27 (56.3)
GLP-1RA, n (%)	16 (33.3)

The data are presented as the means ± standard deviations (SDs), medians (interquartile ranges, IQRs) or numbers (%). BMI: body mass index; SBP: systolic blood pressure; DBP: diastolic blood pressure; UACR: urinary albumin/creatinine ratio; Urinary β2-MG: urinary β2-microglobulin; eGFR: estimated glomerular filtration rate; HbA1c: glycated haemoglobin; TG: triglycerides; TC: total cholesterol; LDL-C: low-density lipoprotein cholesterol; ALT: alanine aminotransferase; AST: aspartate aminotransferase; RASi: renin‒angiotensin system inhibitor; SGLT2i: sodium‒glucose cotransporter 2 inhibitor; GLP-1RA: glucagon-like peptide-1 receptor agonist.

### 3.2 Effectiveness Analysis3.2.1 Differences in the UACR levels and urinary β2-MG levels before and after treatment with finerenone

**Fig 1:**
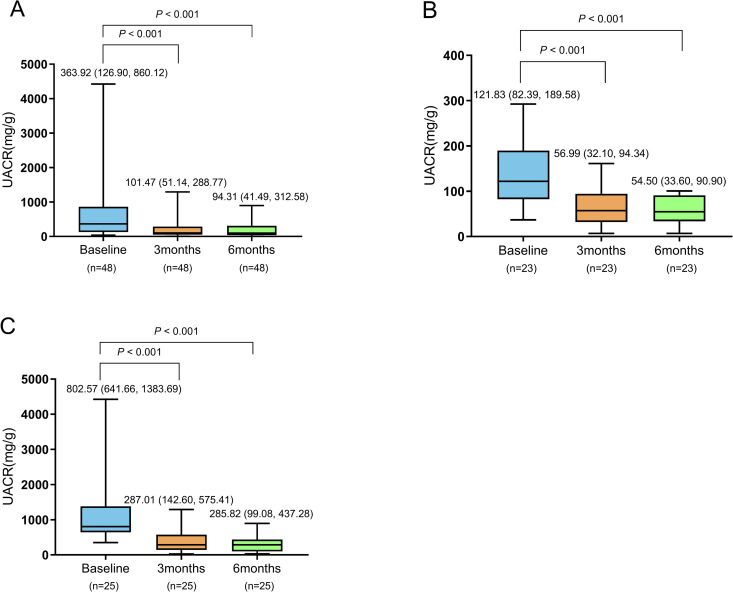
Effects of finerenone on the UACR in all patients. **(A)**, patients with microalbuminuria **(B)**, and patients with macroalbuminuria **(C)**. UACR: urinary albumin/creatinine ratio.

**Fig 2:**
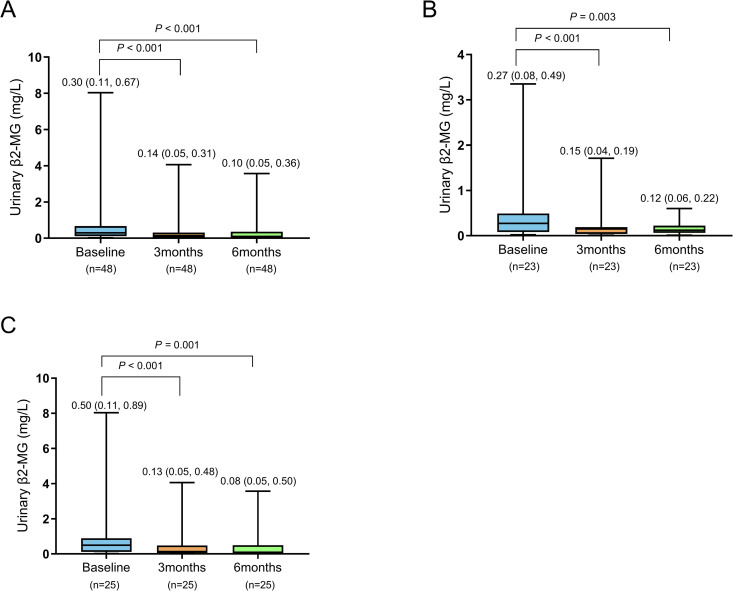
Effects of finerenone on the urinary β2-MG levels in all patients. **(A)**, patients with microalbuminuria **(B)**, and patients with macroalbuminuria **(C)**. Urinary β2-MG: urinary β2-microglobulin.

Compared with baseline, all the patients showed a significant reduction in the UACR and urinary β2-MG levels after 3 and 6 months of treatment with finerenone (**P* *< 0.001) (Table 2, Figs 1–2).

**Table 2:** Changes in the UACR and urinary β2-MG levels compared with baseline values over time.

Variables	Baseline	3 months	*P* value	6 months	*P* value
All patients (n = 48)					
UACR (mg/g)	363.92 (126.90, 860.12)	101.47 (51.14, 288.77)	< 0.001	94.31 (41.49, 312.58)	< 0.001
Urinary β2-MG (mg/l)	0.30 (0.11, 0.67)	0.14 (0.05, 0.31)	< 0.001	0.10 (0.05, 0.36)	< 0.001
Microalbuminuria group (n = 23)					
UACR (mg/g)	121.83 (82.39, 189.58)	56.99 (32.10, 94.34)	< 0.001	54.50 (33.60, 90.90)	< 0.001
Urinary β2-MG (mg/l)	0.27 (0.08, 0.49)	0.15 (0.04, 0.19)	< 0.001	0.12 (0.06, 0.22)	0.003
Macroalbuminuria group (n = 25)					
UACR (mg/g)	802.57 (641.66, 1383.69)	287.01 (142.60, 575.41)	< 0.001	285.82 (99.08, 437.28)	< 0.001
Urinary β2-MG (mg/l)	0.50 (0.11, 0.89)	0.13 (0.05, 0.48)	< 0.001	0.08 (0.05, 0.50)	0.001

The data are represented as the medians (IQRs). The Wilcoxon signed rank sum test was used to compare the data before and after treatment. A *p* value of < 0.05 was considered to indicate a significant difference. UACR: urinary albumin/creatinine ratio; Urinary β2-MG: urinary β2-microglobulin.

The UACR and urinary β2-MG levels of the patients in both the microalbuminuria subgroup and macroalbuminuria subgroup were significantly decreased after 3 and 6 months of treatment with finerenone compared with baseline (**P* *< 0.05) (Table 2, Figs 1–2). Moreover, after 3 and 6 months of treatment, the rates of decrease in the UACR in the macroalbuminuria subgroup were significantly greater than those in the microalbuminuria subgroup (**P* *< 0.05). However, there was no significant difference in the rate of decrease in the urinary β2-MG levels between the two subgroups after 3 and 6 months of treatment (**P* *> 0.05) (Table 3).

**Table 3:** Comparison of the rates of decline in the UACR and urinary β2-MG levels between the two subgroups.

	Microalbuminuria group (n = 23)	Macroalbuminuria group (n = 25)	*P* value
3 months			
Rate of UACR decline (%)	48.37 ± 27.22	64.26 ± 26.84	0.048
Rate of urinary β2-MG decline (%)	40.74 (21.05, 70.18)	49.44 (2.50, 81.23)	0.804
6 months			
Rate of UACR decline (%)	50.99 ± 28.19	71.11 ± 20.26	0.006
Rate of urinary β2-MG decline (%)	26.32 (10.45, 76.00)	55.54 (10.10, 83.03)	0.427

The means ± SDs and medians (IQRs) are presented for normally and nonnormally distributed data, respectively. Normally distributed data were compared between groups with Student’s t test. Nonnormally distributed data were compared between groups with the Mann‒Whitney U test. A *p* value of < 0.05 was considered to indicate a significant difference. UACR: urinary albumin/creatinine ratio; Urinary β2-MG: urinary β2-microglobulin.

#### 3.2.2 Differences in the IL-1β, TNF-α, IFN-γ and IL-10 levels before and after treatment with finerenone.

All the patients exhibited significant decreases in the IL-1β, TNF-α, and IFN-γ levels after 3 and 6 months of treatment with finerenone(**P* *< 0.05), whereas there was no significant difference in the IL-10 levels, compared with those at baseline (**P* *> 0.05) (Table 4).

**Table 4:** Changes in inflammatory cytokine levels compared with baseline values in all patients over time.

Variables	Baseline(n = 48)	3 months (n = 48)	*P* value	6 months (n = 48)	*P* value
IL-1β (pg/ml)	2.33 (1.48, 3.20)	1.18 (0.75, 2.17)	< 0.001	1.12 (0.67, 1.56)	< 0.001
TNF-α (pg/ml)	2.64 (1.90, 3.23)	2.00 (1.30, 2.84)	0.001	1.28 (0.92, 2.21)	< 0.001
IFN-γ (pg/ml)	2.20 (1.58, 2.99)	1.46 (1.06, 2.24)	< 0.001	1.14 (0.71, 1.68)	< 0.001
IL-10 (pg/ml)	3.28 (1.93, 4.64)	3.54 (2.42, 4.64)	0.251	3.16 (2.23, 4.08)	0.774

The data are represented as the medians (IQRs). The Wilcoxon signed rank sum test was used to compare the data before and after treatment. A *p* value of < 0.05 was considered to indicate a significant difference. IL-1β: interleukin-1β; TNF-α: tumour necrosis factor-α; IFN-γ: interferon-γ; IL-10: interleukin-10.

### 3.3 Analysis of other biochemical indicators

After treatment, the levels of HbA1c, TG, TC, and LDL-C in all the patients were significantly lower than those at baseline (**P* *< 0.05) (Table 5). However, the HbA1c, TG, TC, and LDL-C levels in all the patients after 6 months of treatment were not significantly different from those after 3 months of treatment (**P* *> 0.05) (Table 6).

**Table 5:** Changes in blood pressure, HbA1c, blood lipid and liver function-related indicator values compared with baseline values in all patients over time.

Variables	Baseline (n = 48)	3 months (n = 48)	*P* value	6 months (n = 48)	*P* value
SBP (mmHg)	127.60 ± 9.06	127.88 ± 8.76	0.682	127.40 ± 8.27	0.710
DBP (mmHg)	81.77 ± 7.93	81.15 ± 7.45	0.196	81.25 ± 7.83	0.311
HbA1c (%)	8.52 ± 1.96	6.92 ± 1.14	<0.001	6.69 ± 1.00	<0.001
TG (mmol/l)	2.17 (1.41, 2.96)	1.49 (1.03, 2.52)	0.002	1.69 (1.12, 2.55)	0.110
TC (mmol/l)	4.90 ± 1.27	4.20 ± 0.98	0.001	4.25 ± 1.07	0.004
LDL-C (mmol/l)	2.85 ± 0.93	2.52 ± 0.61	0.026	2.50 ± 0.68	0.025
ALT (u/L)	21.30(16.18, 29.15)	21.60(16.85, 27.48)	0.817	19.00(16.20, 31.55)	0.743
AST (u/L)	19.75(15.83, 29.00)	20.05(16.40, 23.18)	0.368	20.25(17.03, 22.80)	0.369

The means ± SDs and medians (IQRs) are presented for normally and nonnormally distributed data, respectively. The paired t test was used to compare the normally distributed data before and after treatment. The Wilcoxon signed rank sum test was used to compare the nonnormally distributed data before and after treatment. A *p* value of < 0.05 was considered to indicate a significant difference. SBP: systolic blood pressure; DBP: diastolic blood pressure; HbA1c: glycated haemoglobin; TG: triglyceride; TC: total cholesterol; LDL-C: low-density lipoprotein cholesterol; ALT: alanine aminotransferase; AST: aspartate aminotransferase.

**Table 6:** Changes in blood pressure, HbA1c, blood lipids and liver function-related indicator values from 3 months to 6 months of treatment in all patients.

Variables	3 months (n = 48)	6 months (n = 48)	*P* value
SBP (mmHg)	127.88 ± 8.76	127.40 ± 8.27	0.435
DBP (mmHg)	81.15 ± 7.45	81.25 ± 7.83	0.730
HbA1c (%)	6.92 ± 1.14	6.69 ± 1.00	0.052
TG (mmol/l)	1.49 (1.03, 2.52)	1.69 (1.12, 2.55)	0.642
TC (mmol/l)	4.20 ± 0.98	4.25 ± 1.07	0.658
LDL-C (mmol/l)	2.52 ± 0.61	2.50 ± 0.68	0.806
ALT (u/L)	21.60(16.85, 27.48)	19.00(16.20, 31.55)	0.061
AST (u/L)	20.05(16.40, 23.18)	20.25(17.03, 22.80)	0.458

The means ± SDs and medians (IQRs) are presented for normally and nonnormally distributed data, respectively. The paired t test was used to compare the normally distributed data from different treatment times. The Wilcoxon signed rank sum test was used to compare the nonnormally distributed data from different treatment times. A *p* value of < 0.05 was considered to indicate a significant difference. SBP: systolic blood pressure; DBP: diastolic blood pressure; HbA1c: glycated haemoglobin; TG: triglyceride; TC: total cholesterol; LDL-C: low-density lipoprotein cholesterol; ALT: alanine aminotransferase; AST: aspartate aminotransferase.

### 3.4 Safety analysis

The eGFRs of all the patients decreased after 1 month of treatment compared with baseline (**P* *< 0.05), rebounded after 3 and 6 months of treatment, with no significant difference relative to the baseline levels, and remained stable (**P* *> 0.05) (Table 7, Table 8). After 1 month, 3 months, and 6 months of treatment, the serum potassium levels of all the patients were significantly greater than the baseline levels (**P* *< 0.001) (Table 7). However, the serum potassium levels of all the patients after 3 and 6 months of treatment were not significantly different from those after 1 month of treatment (**P* *> 0.05) (Table 8). There were no significant changes in blood pressure, ALT, or AST compared with baseline in any of the patients after treatment (**P* *> 0.05) (Table 5). We observed that 2 patients discontinued treatment with finerenone due to hyperkalemia resulting in less than 3 months of treatment. During the study period, none of the patients reported hypotension, allergies, liver dysfunction, breast pain, or male breast development.

**Table 7:** Changes in the eGFRs and serum potassium levels compared with baseline values in all patients over time.

Variables	Baseline (n = 48)	1 month (n = 48)	3 months (n = 48)	6 months (n = 48)
eGFR (ml/min/1.73㎡)	97.79(82.22, 111.13)	88.55(60.54, 105.36)^*^	91.34(78.47, 109.23)	96.04(79.12, 108.97)
Serum potassium (mmol/l)	3.95 ± 0.34	4.26 ± 0.36^***^	4.31 ± 0.31^***^	4.21 ± 0.37^***^

The means ± SDs and medians (IQRs) are presented for normally and nonnormally distributed data, respectively. The paired t test was used to compare the normally distributed data before and after treatment. The Wilcoxon signed rank sum test was used to compare the nonnormally distributed data before and after treatment. A *p* value of < 0.05 was considered to indicate a significant difference. ^*^p value < 0.05. ^***^p value < 0.001. eGFR: estimated glomerular filtration rate.

**Table 8:** Changes in the eGFRs and serum potassium levels compared with values at the end of 1 month of treatment in all patients over time.

Variables	1 month (n = 48)	3 months (n = 48)	*P* value	6 months (n = 48)	*P* value
eGFR (ml/min/1.73㎡)	88.55(60.54, 105.36)	91.34(78.47, 109.23)	0.218	96.04(79.12, 108.97)	0.063
Serum potassium (mmol/l)	4.26 ± 0.36	4.31 ± 0.31	0.420	4.21 ± 0.37	0.401

The means ± SDs and medians (IQRs) are presented for normally and nonnormally distributed data, respectively. The paired t test was used to compare the normally distributed data from different treatment times. The Wilcoxon signed rank sum test was used to compare the nonnormally distributed data from different treatment times. A *p* value of < 0.05 was considered to indicate a significant difference. eGFR: estimated glomerular filtration rate.

## 4. Discussion

Asian DKD patients have a greater risk of kidney disease progression. A cross-sectional study on the global prevalence of microalbuminuria [19] revealed that the prevalence of elevated urinary albumin levels and the risk of kidney disease progression in type 2 diabetes patients in Asia are greater than those in other regions of the world. Recent clinical trials provided robust evidence that finerenone significantly improved kidney and cardiovascular AEs in patients with type 2 diabetes and CKD [17,18]. However, the benefits and risks of finerenone treatment in real-world settings need to be evaluated. Some prospective studies are ongoing to evaluate the effectiveness and safety of finerenone in the treatment of DKD in real-world practices [20].

Albuminuria is an independent and reliable marker for CKD [21]. In the FIGARO-DKD trial [18], the patients in the finerenone group presented a significant 32% reduction in the UACR after 4 months of treatment compared with the patients in the placebo group. In the FIDELIO-DKD trial [17], compared with the placebo group, the finerenone group presented a 31% decrease in the UACR after 4 months of treatment compared with baseline, and this decreasing trend was maintained after 4 months. Post hoc analysis of clinical trials [22,23] showed that early intervention in reducing albuminuria (> 30%) was associated with a reduced risk of CKD progression. The results of our study showed that after 3 months of treatment with finerenone, the UACR levels of all patients were significantly decreased compared with those at baseline. And on the basis of stable blood glucose control, the UACR levels after 6 months of treatment still showed significant decreases compared to baseline, with an average UACR decrease rate of 50.99% in the microalbuminuria group and 71.11% in the macroalbuminuria group. Compared with previous studies, this higher rate of UACR decline might have been due to different pharmacological mechanisms. In our study, some patients have applied RAS inhibitors and/or SGLT2 inhibitors and/or GLP-1 receptor agonists. RASi, SGLT2i, and finerenone all have the effect of reducing proteinuria and delaying the progression of CKD. RASi, SGLT2i, and finerenone can serve as the three main pillars for treating DKD, forming multiple combination therapy protocols for DKD [24,25], while GLP-1RA may be a potential fourth pillar for treating DKD [26]. Meanwhile, our clinical data showed that the UACR reduction rate in the macroalbuminuria group were greater than those in the microalbuminuria group after 3 and 6 months of treatment with finerenone. Previous studies [21] have shown that the improvement in renal composite endpoint incidence in patients with a baseline UACR ≥ 300 mg/g was greater than that in patients with a baseline UACR between 30 and <300 mg/g. These findings indicate that after treatment with finerenone, patients with macroalbuminuria potentially experience greater benefits.

In addition to glomerular injury, renal tubular injury is an important pathophysiological manifestation of diabetic nephropathy [27]. Urinary β2-MG levels are used as a biomarker of renal tubular injury. A risk assessment of 305 patients with biopsy-confirmed diabetic nephropathy revealed that urinary β2-MG levels were related to the severity of renal tubular interstitial injury and mesangial dilatation in diabetic nephropathy patients [28]. In the early stage of diabetic nephropathy, the urinary β2-MG level may increase, and with the exacerbation of renal insufficiency, the urinary β2-MG level gradually increases. Moreover, there is a significant positive correlation between urinary β2-MG levels and urinary albumin levels [27]. After binding to the overactivated MR, finerenone can delay renal tubular injury by inhibiting proinflammatory and profibrotic factor expression, suppressing oxidative stress, ameliorating mitochondrial dysfunction and mediating other mechanisms [29,30]. In this study, some patients received combinations of RAS inhibitors, SGLT2 inhibitors, and/or GLP-1 receptor agonists, all of which offer protection to renal tubular function [31–33]. The results of this study showed that after 3 and 6 months of finerenone treatment, the urinary β2-MG levels of all the patients significantly decreased compared with those at baseline. These findings indicate that combining finerenone with RAS inhibitors, SGLT2 inhibitors, and/or GLP-1 receptor agonists may ameliorate renal tubular injury in patients with DKD.

Various proinflammatory factors are associated with the progression of DKD. Previous animal experiments revealed that the mRNA expression of IL-1β and TNF-α in the renal cortex of diabetic rats was significantly greater than that in the renal cortex of nondiabetic rats [34]. IL-1 can increase the permeability of vascular endothelial cells, leading to increased urinary protein excretion. Moreover, TNF-α can induce cell apoptosis and necrosis, increase reactive oxygen species production, and increase albumin filtration, leading to kidney damage [35,36]. Previous studies have shown that IFN-γ play an important role in the progression and late stages of DKD, up to end-stage renal disease [8]. As a type of MRA, finerenone can reduce the gene expression of proinflammatory factors [6]. After the administration of finerenone to diabetes model mice, the levels of proinflammatory cytokines in the perivascular and perirenal adipose tissues were reduced, which ameliorated endothelial cell function and kidney damage [37]. Meanwhile, GLP-1RA, RASi, and SGLT2i can all exert effective anti-inflammatory effects through multiple mechanisms [38–40]. In this study, 54.2% of patients were treated with RAS inhibitors, 56.3% with SGLT2 inhibitors, and 33.3% with GLP-1 receptor agonists. We discovered that the levels of IL-1β, TNF-α and IFN-γ in patients decreased compared with the pretreatment levels. And on the basis of stable blood glucose control, the levels of IL-1β, TNF-α and IFN-γ after 6 months of treatment still showed significant decreases compared to baseline, which indicates that combining finerenone with RAS inhibitors, SGLT2 inhibitors, and/or GLP-1 receptor agonists can exert anti-inflammatory effects by reducing proinflammatory cytokine levels, thereby ameliorating inflammatory responses, protecting the kidneys. IL-10 is an anti-inflammatory factor that is secreted by immune cells, and it can improve the prognosis of kidney diseases through mechanisms such as activating anti-inflammatory responses, regulating immune responses, and reducing renal tissue fibrosis [9]. However, there was no significant difference in the level of IL-10 compared with the pretreatment level, which indicates that finerenone may not have a significant effect on the levels of anti-inflammatory factors.

Previous studies [41] have shown that in the early stages of treatment with finerenone, acute decline in eGFR may occur due to hemodynamic effects. Meanwhile, RASi, SGLT2i also can reduce intraglomerular pressure. However, this current study did not observe a greater renal function deterioration although over half of the patients received a combination therapy of finerenone with RAS inhibitors and/or SGLT2 inhibitors. With a comparable effect of finerenone on eGFR in other studies [17,42,43], eGFR in our study decreased at 1 month of finerenone treatment. This may be due to the intraglomerular pressure reduction caused by the use of finerenone alone or the combination therapy. Even if some patients were treated with RAS inhibitors and/or SGLT2 inhibitors, the eGFR levels after 3 and 6 months of finerenone treatment rebounded to levels that were not significantly different from the baseline level, and then remained stable. Despite this hemodynamic change, vigilance is still warranted to prevent worsening renal function in clinical practice, especially when medications that cause a decrease in intraglomerular pressure are used concomitantly or when hypotension or hypovolemia is present.

Hyperkalemia is currently the confirmed risk in clinical trials involving the use of finerenone [43,44]. In our study, with 54.2% of patients receiving RAS inhibitors in combination, the serum potassium levels were elevated in all patients after 1, 3, and 6 months of treatment compared with those at baseline. This may be related to the increased risk of hyperkalemia caused by the dual inhibition of the renin–angiotensin–aldosterone system. A retrospective real-world study with a high proportion (85.7%) of RAS inhibitors and finerenone combination [45] has shown that the incidence of hyperkalemia (> 5.5 mmol/L) at the end of the 1, 3, and 6 months of treatment was 4.8%, 7.1%, and 4.8%, respectively. And an increase in serum potassium concentration was observed at both the 3 and 6 months of treatment. But in our study, the serum potassium levels at the end of 3 and 6 months of treatment did not further increase compared with that at the end of 1 month of treatment and tended to stabilize. Compared with previous study, the more stable serum potassium concentration in our study may be related to the lower proportion of patients treated with RAS inhibitors in combination. These findings indicate that there may be an increase in the blood potassium concentration during the initial stage of finerenone treatment, especially when finerenone is combined with RAS inhibitors. In the stage of screening research subjects, we found that two patients discontinued finerenone due to hyperkalemia. They were both elderly and complicated by unilateral renal artery occlusion or long-term chronic renal insufficiency. Therefore, during the application of finerenone treatment in real clinical practice, the serum potassium concentration of patients, especially those receiving combined application of RAS inhibitors and those with special factors such as renal artery stenosis and long-term chronic renal insufficiency, should be closely monitored.

This study has several limitations. Firstly, this was a single-centre study with a small sample size (n = 48). Secondly, this study lacked long-term observation of finerenone treatment, randomized grouping comparison between finerenone treatment group and non treatment group, and grouping comparison of combination therapy with different drugs and finerenone. Therefore, it is necessary to conduct a randomized controlled trial with a larger sample size and a longer follow-up time, as well as the observation of the evaluation of the effect after discontinuing the treatment of finerenone, to evaluate the treatment effect of finerenone on diabetic nephropathy patients.

## 5. Conclusion

In real clinical practice, with the assistance of RAS inhibitors, SGLT2 inhibitors, and GLP-1 receptor agonists, finerenone safely and effectively reduces the urinary protein levels in DKD patients and ameliorates glomerular and tubular function, delaying disease progression. Among these patients, those with macroalbuminuria may experience greater benefits. The combination of finerenone with RAS inhibitors, SGLT2 inhibitors, and/or GLP-1 receptor agonists may delay the progression of kidney disease by improving the inflammatory response. Finally, the individualized effect of concomitant medications needs to be carefully evaluated in routine clinical practice.
